# Are we missing something? Different obstructive sleep apnea phenotypes as a possible driver of discrepancies in cognitive recovery after continuous positive airway pressure treatment

**DOI:** 10.1093/sleep/zsad269

**Published:** 2023-10-21

**Authors:** Javier Gomez-Pilar, Gonzalo C Gutiérrez-Tobal, David Gozal, Roberto Hornero

**Affiliations:** Biomedical Engineering Group, University of Valladolid, Valladolid, Spain; Centro de Investigación Biomédica en Red en Bioingeniería, Biomateriales y Nanomedicina, Instituto de Salud Carlos III, Valladolid, Spain; Biomedical Engineering Group, University of Valladolid, Valladolid, Spain; Centro de Investigación Biomédica en Red en Bioingeniería, Biomateriales y Nanomedicina, Instituto de Salud Carlos III, Valladolid, Spain; Joan C. Edwards School of Medicine, Marshall University, Huntington, WV, USA; Biomedical Engineering Group, University of Valladolid, Valladolid, Spain; Centro de Investigación Biomédica en Red en Bioingeniería, Biomateriales y Nanomedicina, Instituto de Salud Carlos III, Valladolid, Spain

In this issue of the journal, D’Rozario et al. [1] provide a very timely quantitative analysis of brain activity measures using high-density electroencephalography (EEG) in patients with obstructive sleep apnea (OSA). This longitudinal study evaluated the efficacy of continuous positive airway pressure (CPAP) treatment to reverse the effects of OSA on specific brain regions and frequency bands, particularly those previously related to deficits in cognitive traits [2]. Additionally, they directly evaluated such changes in cognitive traits using a test battery to assess the potential enhancement in overnight memory consolidation.

To contextualize the significance of this study and its promising results, one first needs to understand the intrinsic relationships between sleep and cognition. An increasing number of studies have focused on the link between sleep and various cognitive traits, revealing a close relationship between them [2, 3]. As a corollary of such findings, it is critical to establish whether patients with conditions closely related to sleep, especially pathologies that directly affect sleep quality, show impaired cognitive processes. This is obviously applicable to diseases such as OSA, a highly disruptive condition that imposes a significant adverse impact on the quality and quantity of restorative sleep.

With nearly 1 billion adults aged 30–69 years worldwide estimated to be affected [4], OSA is primarily characterized by the repetitive collapse of the pharyngeal airway during sleep, resulting in intermittent breathing interruptions categorized as apneas or hypopneas depending on the degree of collapsibility during the event. These apneic events not only lead to intermittent hypoxia and altered systemic blood pressure and cardiac frequencies, which could potentially exert deleterious effects on various end organs, but they also often are accompanied by arousals that disrupt the natural cyclical architecture of sleep. Interestingly, recent investigations have shown an even more complex relationship between OSA and sleep physiology, indicating that its effects on sleep go beyond mere sleep fragmentation [5]. In this regard, patients with OSA experience a reduced quality of life, while potentially increasing the risk of long-term cardiovascular, metabolic, neurocognitive, and behavioral morbidities [6].

In an effort to further understand the impact of OSA on the neurophysiology of patients, cross-sectional studies have examined differences in electroencephalographic (EEG) oscillations. Significant findings have emerged, such as the progressive deceleration of slow oscillations with the severity of OSA [7] or a reduction in slow wave activity in patients with OSA [8]. Considering that the EEG frequency bands linked to these oscillations are intricately associated with sleep spindle generation [9], it is not surprising that some researchers have also identified abnormalities in spindle-related activity in patients living with OSA [10]. Collectively, these findings emphasize the significant role of OSA in inducing alterations in both sleep macro-structure and micro-structure. Given the role of these oscillations in different cognitive traits [2], the characterization of these abnormalities in neural activity during sleep is of noteworthy significance.

The longitudinal study by D’Rozario et al. [1] not only corroborates the deficits in EEG oscillatory patterning in patients living with OSA, but further delves into this issue by investigating the influence of CPAP treatment on 11 adult male participants. The authors analyzed the overnight EEG, which was acquired using a high-density configuration (64 channels). The results suggest the possibility for an optimistic outlook, revealing an extension in the duration of deep sleep stages (REM and N3) at the expense of a significant reduction in light sleep stages (N1 and N2). Moreover, several changes in frequency bands and specific brain regions were identified, encompassing an increase in gamma and delta power during N3 and a reduction in slow spindle activity within fronto-central regions following CPAP treatment. These results highlight the importance of using both high-density EEG and longitudinal study design, the latter complementing the more frequent case–control studies.

Along with these changes in neuronal activity, a series of improvements in different cognitive characteristics related to memory consolidation could be expected. That is why the authors carried out a test battery to evaluate the neurobehavioral performance before and after sleep in both baseline and after CPAP treatment. Unfortunately, the results did not demonstrate enhancements in cognitive functions after treatment within important domains such as executive function, working memory, sustained attention, and overnight declarative memory consolidation. These findings are in line with a previous study in older adults, in which no positive associations of slow wave activity or spindle activity were found with overnight memory performance [11]. However, they diverge from other observational studies in which improvements in procedural and declarative memory after CPAP were reported [12, 13]. These a priori negative findings, far from diminishing the relevance of the study, may actually reveal possible causes of these discrepancies. For example, among the numerous potential factors contributing to these disparities may stem from methodological differences, such as the sample size, the study design (longitudinal or cross-sectional studies), or the use of a significantly different number of EEG electrodes, among others. Nevertheless, another crucial aspect to be considered is the coexistence of different OSA phenotypes in the same population under study. In support of this idea, significant heterogeneity in patient response and adherence to treatment has been previously reported [14].

In this context, different phenotypes have been identified within adult patients living with OSA by using clinical and sociodemographic characteristics [15, 16]. Notably, 23 000 adults were involved in the definition of 11 OSA phenotypes [16] each of which may respond disparately to various treatment interventions. Although pediatric OSA is not as extensively investigated, phenotyping has been also approached by using information from different overnight PSG-derived signals. This is the case of reports that analyzed EEG and ECG to define different OSA phenotypes in children focused on neurocognitive and cardiovascular functioning [6, 17]. In a similar way, a new study revealed different metabolic (MetS) and inflammatory (C-reactive protein) profiles causally attributable to the presence of more apneas and hypopneas in children, respectively [18]. Therefore, these findings are coherent with the idea of the existence of different OSA phenotypes, both in adults and children, which in turn may dictate the degree of reversibility when treatment is implemented.

**Figure 1.**
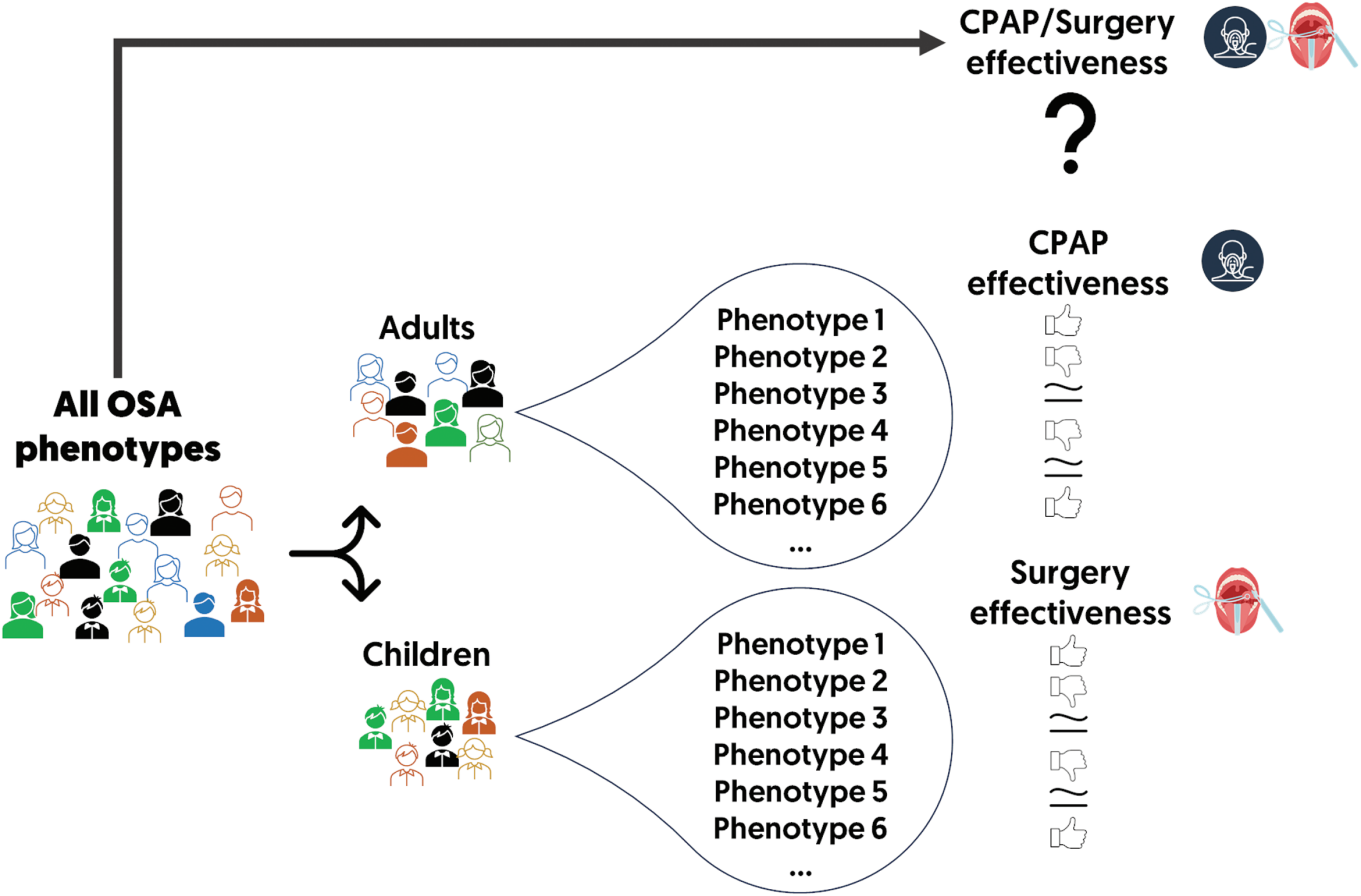
Schema of the potential benefits of the identification of different phenotypes in patients with obstructive sleep apnea prior to treatment.

In summary, divergent OSA phenotypes could be the underlying cause for the heterogeneous responses observed in response to therapy across multiple studies, including divergent cognitive improvements post-treatment (see Figure 1). Consequently, a thorough characterization of OSA and its various phenotypes is of the utmost importance to prevent erroneous conclusions. This detailed understanding is essential for an independent evaluation of the genuine response to treatment for each distinct phenotype, shedding light on the intricate nuances of tailored OSA treatments.
